# A meta-analysis of kidney microarray datasets: investigation of cytokine gene detection and correlation with rt-PCR and detection thresholds

**DOI:** 10.1186/1471-2164-8-88

**Published:** 2007-03-30

**Authors:** Walter D Park, Mark D Stegall

**Affiliations:** 1 Department of Surgery, Mayo Clinic College of Medicine, Rochester, MN, USA

## Abstract

**Background:**

Microarrays provide a means to simultaneously examine the gene expression of the entire transcriptome in a single sample. Many studies have highlighted the need for novel software and statistical approaches to assess the measured gene expression. Less attention has been directed toward whether genes considered undetectable by microarray can be detected by other strategies or whether these genes can provide accurate gene expression determinations. In the kidney this is a concern for genes such as cytokines which dramatically influence the immune response but are often considered low abundance genes produced by a small number of cells.

**Results:**

Using both publicly available and our own microarray datasets we analyzed the detection p-value and detection call values for 81 human kidney samples run on the U133A or U133Plus2.0 Affymetrix microarrays (Affymetrix, Santa Clara, CA). For the cytokine genes, the frequency of detection in each sample group (normal, transplant and renal cell carcinoma) was examined and revealed that a majority of cytokine related genes are not detectable in human kidney by microarray. Using a subset of 29 Mayo transplant samples, a group of seven transplant-related cytokines and eight non-cytokine genes were evaluated by real-time PCR (rt-PCR). For these 15 genes we compared the impact of decreasing microarray detection frequency with the changes in gene expression observed by both microarray and rt-PCR. We found that as microarray detection frequency decreased the correlation between microarray and rt-PCR data also decreased.

**Conclusion:**

We conclude that, when analyzing microarray data from human kidney samples, genes generally expressed at low abundance (i.e. cytokines) should be evaluated with more sensitive approaches such as rt-PCR. In addition, our data suggest that the use of detection frequency cutoffs for inclusion or exclusion of microarray data may be appropriate when comparing microarray and rt-PCR gene expression data and p-value calculations.

## Background

The recent completion of the human genome project, improvements in gene level annotation and microarray technology have led to a rapid increase in the number of whole genome microarray based studies for researchers interested in understanding the underlying etiology of various human conditions. However, there remain several significant questions to answer with regard to microarray data acquisition and analyses, including the accurate determination of signal intensity, development of optimal analytical strategies and detection limit thresholds.

The most common oligonucleotide microarray platform is made by Affymetrix Inc. and uses 25-mer probes to target gene sequences. Approximately 22 different probes, equally divided between paired perfect match (PM) and mismatch (MM) probes, make up a probeset which is designed to target a specific gene. Fluorescent data from each PM-MM pair is analyzed by the Affymetrix MAS 5.0 software and a single value for signal intensity, detection p-value and detection call are generated for each probeset. To determine the best method for analyzing the data, multiple software programs and statistical approaches have been developed [1-6]. However, there continues to be much debate regarding the inclusion of both PM and MM probe signal intensities to provide the most accurate measures of expression [7].

Additional interpretation of Affymetrix microarray data frequently includes filtering the data on the basis of the calculated detection p-value from which a detection call of present, marginal or absent is made for each probeset. Often this information is incorporated into downstream analysis strategies by calculating the percent present (%P) or detection frequency for a probeset based on all of the samples in a study and omitting those probesets that fail to reach the threshold. A recent study explored the effect of filtering using a detection frequency cutoff and suggested that filtering ≥ 50% %P may improve downstream analysis [8]. However, typically only 30–40% of probesets are considered detectable in any human tissue sample [9]. Therefore, the decision to include a detection frequency cutoff will dramatically alter the number of probesets used in downstream analysis.

It is important to note that the use of a detection frequency filter presumes that genes which are considered undetectable by the microarray are not actually present in the sample. However, given the lack of sensitivity of microarrays, transcripts of low abundance within a sample may not be detected [10]. One such family of genes are the cytokines which may only be expressed by a small fraction of the cells in human organs, such as the kidney, or by a small number of infiltrating immune cells. Interestingly, several members of the cytokine gene family have been found significantly altered in samples of human renal cell carcinoma (RCC) and kidney transplant rejection [11-15]. In general, these associations have been made using real-time PCR (rt-PCR) and not microarray. To date no report has assessed the detectability of cytokines with microarrays in human kidney under a variety of physiological conditions.

In this study we used a meta-analysis of publicly available Affymetrix datasets to explore the detection frequency of cytokine genes in the human kidney. We then selected a subset of genes with high and low detection frequencies and performed follow-up rt-PCR on a group of kidney transplant samples. Finally, we performed a comparison of gene expression changes observed by microarray and rt-PCR to assess the accuracy of data obtained from high and low detection frequency genes.

## Results

### Cytokine detection frequency in kidney

To assess the frequency with which cytokine genes are identified as present or absent in human kidney we performed a meta-analysis of publicly available (GEO) Affymetrix microarray datasets related to human kidney tissue. Data from the human U133A and U133Plus2.0 was selected as the sequence used for each probeset is identical on each chip, with only the feature size and array density being different. In addition we used data from 30 human kidney samples from our group. In total there were 81 samples for which both Affymetrix derived detection p-values and detection calls had been made. For simplification the samples were categorized into one of three groups: normal kidneys (n = 15), transplanted kidneys (n = 36) and RCC (n = 30). The thresholds for determining detection calls, the default set by Affymetrix, were the same for all samples.

According to the 4-16-06 Affymetrix annotation file for the U133Plus2.0, there are 393 probesets identified as being related to the "cytokine activity" Gene Ontology classifier (GO:0005125), including many chemokine, interleukin and interferon genes. We also included 5 probesets for the pro-inflammatory genes TGF-β_1 _and FoxP3 which were previously associated with post kidney transplant outcomes using rt-PCR [15,16]. Of the 398 total probesets, 244 (61.3%) had a detection frequency less than 25%.

Table 1 shows a subset of the cytokine probesets, selected primarily because they are designed to detect specific interleukin or interferon genes. The name of the gene and detection rate for all of the samples is provided as well as the breakdown of the detection frequency in the various types of samples. Importantly, using the gene name as an identifier, it is clear that multiple probesets are present for many genes but that these replicate probesets may not always generate similar detection rates. For example, the Interleukin 1 alpha gene has two probesets, one with an overall detection rate of 63% while the other is just 1%. It is unclear what the cause of such variation is but sequence variants or poor probe design seem like plausible explanations.

### Detection by rt-PCR versus microarray

Using 29 of the 30 human Mayo kidney transplant samples, we compared the detection frequency of cytokines for both microarray and rt-PCR. We selected a small group of cytokines that had been previously described using rt-PCR to be associated with various clinical post-transplant events but were represented by only one probeset (Table 2). We also looked for genes that had multiple undetectable probesets from the meta-analysis (TGFβ_1 _and FoxP3). Finally, we included the IFNγ-receptor 1 (IFNγ-R1) gene to serve as a positive control. There are three probesets for this gene on the U133Plus2.0, all of which were detected in 100% of human kidney samples.

To rule out incompatible probe sequence design issues we performed a BLAST search of the Affymetrix consensus sequence for each probeset. We found that, although there are three probesets attributed to the FoxP3 gene on the microarray, only one of the probesets has 100% homology to the FoxP3 Reference Sequence number (NCBI: NM_014009). The other two probesets bind to the JM2 sequence (NCBI: AJ005891) and do not share 100% homology to the same FoxP3 sequence. A similar probe design issue applies to one of the three IFNγ-R1 gene probesets which actually binds to the intron region of the DNA sequence for the gene and is therefore unsuitable for mRNA analysis. This finding has been corroborated elsewhere [17].

Once the probeset sequence was confirmed we performed a comparison of the detection sensitivity between the two platforms using separate aliquots of RNA from each sample. Seventy ng of total RNA was used for microarray processing whereas each rt-PCR reaction used the equivalent of 10 ng total RNA (1 μg RNA aliquot was cDNA amplified and diluted 1:100). For the genes TNFα, IFNγ, IL-10, TGF-β_1 _and FoxP3, none of the Mayo transplant samples were detectable using the microarray (detection p-values ranged from 0.562 to 0.9536) whereas using rt-PCR all of these genes were detectable for all samples tested. Likewise IL-6, selected because it was detectable in 70% of the microarray samples, and the positive control, IFNγ-R1, were detectable by rt-PCR for all samples. In addition, although a relative standard curve was employed to monitor detection by rt-PCR, we did record the minimum and maximum Ct for each gene target (Table 2). There does not appear to be a strong correlation between the microarray detection p-value and Ct value. For example, TGF-β_1 _had one of the highest detection p-values and one of the lowest Max Ct values.

Further evidence for increased detection sensitivity of rt-PCR over microarrays is provided by data from an additional eight non-cytokine genes. Using rt-PCR all samples were considered detectable by rt-PCR even though the detection frequencies ranged from 42–100%.

### Gene expression differences

Using the same transplant samples as above we compared the impact of decreased microarray detection frequencies with the changes in gene expression observed by both microarray and rt-PCR. Changes in gene expression were examined using two distinct patient groups. The first group contained patients that had normal renal pathology at both the time of transplant and subsequent 12 month protocol biopsy (TxNormal [0–12]). The second patient group had normal pathology at the time of transplant but showed evidence of fibrosis on the 12 month protocol biopsy (TxFibrosis [0–12]). The microarray data from both patient groups was analyzed for the changes in gene expression that occurred from the time of transplant to 12 months. A comparison of the microarray and rt-PCR gene expression data was performed using the same 15 genes described above (Table 3). All genes were detected by rt-PCR in each sample, whereas by microarray the samples showed a continuum of detection frequency. As can be seen in Table 3, the five cytokine genes considered undetectable by microarray show no correlation with the rt-PCR data, both in terms of the determination of up/down-regulation and significance. In stark contrast, the high detection frequency genes showed an excellent correlation between microarray and rt-PCR data. Nineteen of the twenty comparisons, including instances of up/down-regulation, correlate. The only instance where correlation did not occur was when the detection frequency was <50%.

## Discussion

Using a meta-analysis of publicly available kidney microarray datasets we were able to show that the Affymetrix platform fails to detect a number of human cytokine genes. For a subset of these samples we were able to show that cytokine genes considered undetectable by microarray were detectable using rt-PCR. This is a significant finding as cytokines are known to be important peptides that regulate innate host defenses and the immune system [18]. In fact, it is currently believed that the underlying etiology of various human conditions such as cancers or transplant rejections involve the expression of multiple cytokines.

The oncogenesis of RCC, which accounts for approximately 80% of all kidney tumors in adults, has been hypothesized to involve modulated cancer cell expression of inflammatory cytokines [11,19,20]. Indeed, multiple cytokines are currently used as immunotherapies to treat this disease [21]. In addition, various reports have linked either the detection of or increased presence of various cytokine mRNA species with disease progression, typically through the use of rt-PCR [*for review see *[22]]. For example, IFN-γ, interleukins 2, 6, 8 and 10 and TNFα have all been identified as detectable or significantly altered in human RCC tumors [11-13,23]. Our meta-analysis showed that in 30 RCC samples, only interleukin 8 had a probeset that was detected in more than half of the samples, with interleukins 2 and 10 considered detectable in less than 10% of the samples.

In human kidney transplantation several reports have also linked the gene expression of various cytokines with transplant events such as acute and chronic rejection [14,15]. Similar to RCC, these links were made using single gene strategies such as rt-PCR, yet the majority of these cytokines were not observed in any of the transplant samples via microarray. For example, TGF-β_1 _has been shown in multiple studies to be linked with chronic rejection but was considered absent in all 36 transplant microarray samples.

The lack of detection of relevant cytokine genes could be related to several issues including improper gene annotation at both the probeset and probe level. Gene annotation is the assignment of gene level information based on the probeset sequences, such as the name and symbol of the gene being interrogated and its general function. As knowledge of a probeset sequence increases it is possible that a probeset will be designated a different gene name. Perez-Iratxeta et al. explored this idea using Affymetrix murine microarrays and showed that as many as 30% (13699/45000) of the probesets were reassigned to a different gene at least once over a two year period [24]. We examined the four quarterly U133Plus2.0 Affymetrix annotation files from 2005 and found the amount of gene assignment changes between probesets ranged between 7–15% each quarter (*Data not shown*). However, none of the cytokine probesets subjected to rt-PCR for this study had a change in gene assignment during that time.

Individual probe sequences can also impact gene annotation. For some probesets not all probes interrogate the mRNA sequence for a single gene. We found this was the case for three of the fourteen cytokine probesets manually examined by BLAST. Two of the probesets have probes that appear to bind a different gene and one probeset has probes that bind to the intron region of the target gene. Recently Harbig et al. used the BLAST program to interrogate the sequence of all the U133Plus2.0 probes against the human genome [17]. Their analyses showed that approximately 37% of the probesets on the U133Plus2.0 should be assigned to a different gene. If we compare the re-assigned data to our list of cytokine probesets we find that approximately 75% (293/398) would have the same gene assignment. However, the percentage of probesets with a detection frequency less than 25% would not change.

Another explanation for the lack of detection could be that microarrays are generally considered less sensitive overall than rt-PCR platforms. This decreased sensitivity could be attributed to several factors including the variation in platform chemistries or probe sequence lengths and designs. However, few groups have compared the detection and correlation of microarrays to rt-PCR in a systematic manner in human samples [25-27]. Wang et al generated rt-PCR data on 1,375 human genes and compared this data to that generated by Applied Biosystems and Agilent microarrays. Using the rt-PCR data as the "ground truth", they found that microarrays did not detect between 24 and 29% of the genes detected by rt-PCR [26]. These findings compliment our data as wewere able to detect several cytokine genes by rt-PCR that we did not detect using microarray even though the amount of input RNA was higher in the microarray samples. This suggests that microarrays may yield a significant number of false negative detection calls when compared to rt-PCR. In contrast, for probesets considered detected in all samples we have consistently been able to use rt-PCR to detect the gene, suggesting the false positive rate for the detection of moderately expressed genes is relatively low.

Although taking into account the lack of detection of genes is important in microarray analysis, it is equally important to ensure that only the most accurate measures of gene expression are being examined. Typically this is done by removing the probesets exhibiting signal considered below the threshold of detection. One approach to eliminate these probesets is to use a detection frequency filter and then proceed with statistical analysis on the remaining probesets. Despite its significance, there are few reports that directly discuss what the detection frequency should be or its effect on data accuracy. However, a recent paper by McClintick et al. concluded that restrictive filtering, such as a ≥ 50% %P, greatly improves the reproducibility and the false discovery rate [8]. Our data both corroborates and extends these findings by showing that observed changes in gene expression by microarray correlate very well with rt-PCR when the probeset has a detection frequency of >50% for the samples under consideration. Conversely, when a probeset is not considered detectable in the majority of samples the correlation between rt-PCR and microarray data is quite poor. Similar to our findings, Etienne et al. also reported finding a lower correlation between rt-PCR and raw Affymetrix signal data as the percentage of absent calls increased [28]. The fact that multiple reports have now shown an important correlation between the detection frequency and rt-PCR suggests that this is an important issue to consider when performing microarray analysis.

## Conclusion

Our meta-analysis of publicly available microarray datasets revealed that microarrays failed to detect multiple cytokine genes that have been linked to various human kidney conditions. This lack of detection may be related to incorrect gene annotation or limited assay sensitivity. For a subset of genes, a comparison between detection frequencies using microarrays and rt-PCR platforms showed that rt-PCR is the more sensitive platform. The detection frequency also had an effect on measured changes in gene expression. For genes considered detectable in the majority of microarray samples, the changes in gene expression observed by microarray and rt-PCR had excellent correlation. No correlation was observed between rt-PCR and microarray when the detection frequency dropped below 50%. Therefore, in the human kidney we would recommend using rt-PCR to detect and assess changes in gene expression among genes of low abundance, such as cytokines, rather than microarrays.

## Methods

### Affymetrix microarray overview

For human samples Affymetrix offers two popular high density microarrays: the U133A and the whole genome U133Plus2.0 microarrays. The U133A contains approximately 22,000 probesets while the U133Plus2.0 has even smaller feature size and contains 54,675 probesets representing approximately 24,000 novel gene titles or UniGene IDs. The majority of probesets contain a total of 22 different 25-mer probes, equally divided between paired perfect match (PM) and mismatch (MM) probes. Each MM probe contains a single base pair difference from its PM counterpart, a single base pair change at the center nucleotide, and its fluorescence is intended to represent background non-specific hybridization. Fluorescent data from each PM-MM pair is then analyzed by the Affymetrix MAS 5.0 software and a single value for signal intensity, detection p-value and detection call (among other variables) are generated for each probeset. For our study, the MAS 5.0 default parameters were retained. For a complete description of the MAS 5.0 algorithms and statistical tests please refer to the Affymetrix manuals [29]. Importantly, for each microarray, Affymetrix releases a new annotation file that is updated each quarter and contains a large amount of information, including the gene name, UniGene ID and gene ontology associated with each probeset and probeset sequence [30].

### Public microarray datasets

Microarray datasets were obtained from the publicly available Gene Expression Omnibus (GEO) website [31]. GEO Datasets were queried, filtered and selected on the basis of human kidney and Affymetrix platform data. In addition, to be included in the study the following fields were required to be reported: probeset ID, expression value, detection call and detection p-value. A total of three data series fulfilled these requirements: GSE2004, GSE3297 and GSE2109. All data was generated using MAS 5.0 (Affymetrix, Inc., Santa Clara, CA) with the same detection call cutoffs used to determine presence or absence for each probeset.

### Internal microarray datasets

An additional 30 samples were obtained from kidney transplant patients biopsied using a protocol approved by the Institutional Review Board of the Mayo Foundation and Clinic. Total RNA was extracted from each biopsy in a RNA-only area using a modified and previously optimized (for this type/size of sample) extraction method using TRIzol^® ^Reagent (Invitrogen Corp, Carlsbad, CA) followed by clean-up with RNeasy Mini Kit^® ^(Qiagen Inc., Valencia, CA) [32]. RNA integrity was assessed to ensure RNA quality and the samples were sent to the Mayo Advanced Genomics Technology Center (AGTC) which processes ~1,000 GeneChips each year. The AGTC processed 70 ng of total RNA for all samples using the two-cycle target labeling, hybridization and scanning procedure described in detail in the Affymetrix GeneChip^® ^Expression Analysis Manual (Affymetrix Inc., Santa Clara, CA). The .cel files from this study can be downloaded from the GEO website: GSE7392.

### Changes in gene expression

Using the Mayo microarray data, two groups of transplant patients were analyzed to assess the changes in gene expression observed by microarray. The first patient group (n = 7) had normal pathology both at the time of transplant (baseline) and at 12 months post transplant (TxNormal [0–12]). The second group of patients (n = 8) had normal pathology at the time of transplant and showed evidence of fibrosis at 12 months post transplant (TxFibrosis [0–12]). For each patient group differential gene expression (baseline to 12 months post transplant) was assessed using a PM-only model, wherein only the data from the PM probes was used to estimate the expression level of individual probesets. The PM data from each chip was subjected to FASTLO, a previously described non-linear method of normalization [6]. Significance for each probeset in this case was determined using a generalized linear model with random effects for chip and treatment, analogous to a t-test. P-values were not adjusted for multiple comparisons.

### rt-PCR

To confirm the presence or absence of specific cytokine genes recorded by the microarray, a rt-PCR strategy was employed. Using the Mayo Clinic General Clinical Research Center we utilized rt-PCR to examine target genes and a housekeeping gene (18S) for 29 of the Mayo samples for which at least 1 μg of RNA was available for cDNA synthesis. For each gene, assays containing pre-designed primers and probes designed from the matching Reference Sequence number provided by Affymetrix were purchased from ABI (Applied Biosystems, Foster City, CA). To generate the standard curves, we created a large pool of human RNA that contained 90% human kidney RNA and 10% mononuclear cell RNA. The mononuclear cell RNA was included to ensure that cytokine mRNA would be present in the control RNA pool. Several 1 μg aliquots of control RNA were converted into cDNA and, after testing to confirm equal efficiency, were batched together. A 10-point standard curve was amplified in triplicate along with a triplicate amplification of each sample (10 ng/rxn). For each assay the top curve point was 36 ng control cDNA followed by multiple 2.5-fold dilutions to as low as 0.01 ng/rxn. While not all assays successfully amplified the lowest points in the curve, no curve amplified less than 6 consecutive points. For example, all 10-points amplified for TGF-β_1_, the top 8 amplified for IL-10 and the top 6 for IFNγ. For each assay we assessed the Ct value for each triplicate standard curve point and found that the majority had a %CV <1%. The highest %CV was 2.89%. For a particular gene to be considered detectable in a given sample the Ct value for the sample reactions needed to be above the lowest detected point on the corresponding standard curve. Cytokine gene selection was based on the presence of the gene in one of three categories: a. Undetectable in all Mayo microarray samples: IFNγ (ABI#: Hs99999041_m1), TNFα(Hs00174128_m1), IL-10 (Hs00174086_m1), TGF-β_1 _(Hs00998133_m1) and FoxP3 (Hs00203958_m1). b. Undetectable in some Mayo microarray samples: IL-6 (Hs99999032_m1), c. Detectable in all Mayo microarray samples: IFNγ-R1 (Hs00988304_m1). Using a strategy similar to that noted above 8 additional non-cytokine genes, the majority of which were detected in all microarray samples, were amplified to compare observed changes in gene expression. These genes were GRZMA (Hs00196206_m1), FN1 (Hs00277509_m1), IER3 (Hs00174674_m1), STAT2 (Hs00237139_m1), COL1A1 (Hs00164004_m1), STAT1 (Hs00234829_m1), VCAM1 (Hs00174239_m1) and PSMB8 (Hs00188149_m1).

## List of abbreviations

Advanced Genomics Technology Center (AGTC)

Gene Expression Omnibus (GEO)

Mismatch (MM)

Percent present (%P)

Perfect Match (PM)

Real-time PCR (rt-PCR)

Renal cell carcinoma (RCC)

## Authors' contributions

WP was responsible for designing, analyzing, collating and interpreting the data and drafting the paper. MS contributed to the design of the study and read the manuscript and provided comments. All authors read and approved the final manuscript.

## Figures and Tables

**Table 1:** Microarray detection frequencies for various cytokines in human kidney samples.

	Probe Set ID	Gene Name	Total samples^a^	%P ALL	%P Normal (n = 15)	%P Transplant (n = 36)	%P Cancer (n = 30)
1	224211_at	Forkhead box P3 (FoxP3)	60	0%		0%	0%
2	221333_at	Forkhead box P3 (FoxP3)	81	0%	0%	0%	0%
3	221334_s_at	Forkhead box P3 (FoxP3)	81	7%	0%	0%	20%
4	202727_s_at	Interferon gamma receptor 1 (IFNγ-R1)	81	100%	100%	100%	100%
5	242903_at	Interferon gamma receptor 1 (IFNγ-R1)	60	100%		100%	100%
6	211676_s_at	Interferon gamma receptor 1 (IFNγ-R1)	81	99%	100%	97%	100%
7	201642_at	Interferon gamma receptor 2	81	91%	80%	89%	100%
8	210354_at	Interferon, gamma (IFNγ)	81	10%	0%	3%	23%
9	208200_at	Interleukin 1, alpha	81	63%	33%	53%	90%
10	210118_s_at	Interleukin 1, alpha	81	1%	0%	0%	3%
11	39402_at	Interleukin 1, beta	81	38%	33%	47%	30%
12	205067_at	Interleukin 1, beta	81	6%	20%	3%	3%
13	207849_at	Interleukin 2	81	4%	7%	6%	0%
14	207906_at	Interleukin 3	81	0%	0%	0%	0%
15	207539_s_at	Interleukin 4	81	0%	0%	0%	0%
16	207538_at	Interleukin 4	81	0%	0%	0%	0%
17	207952_at	Interleukin 5	81	0%	0%	0%	0%
18	205207_at	Interleukin 6 (IL-6)	81	60%	73%	64%	50%
19	206693_at	Interleukin 7	81	64%	40%	64%	77%
20	241808_at	Interleukin 7	60	72%		42%	100%
21	202859_x_at	Interleukin 8	81	65%	53%	50%	90%
22	211506_s_at	Interleukin 8	81	14%	20%	0%	27%
23	208193_at	Interleukin 9	81	1%	0%	0%	3%
24	207433_at	Interleukin 10 (IL-10)	81	4%	7%	0%	7%
25	206924_at	Interleukin 11	81	0%	0%	0%	0%
26	206926_s_at	Interleukin 11	81	0%	0%	0%	0%
27	207844_at	Interleukin 13	81	0%	0%	0%	0%
28	203085_s_at	Transforming growth factor, beta 1 (TGF-β_1_)	81	49%	67%	0%	100%
29	203084_at	Transforming growth factor, beta 1 (TGF-β_1_)	81	1%	0%	0%	3%
30	207113_s_at	Tumor necrosis factor, alpha (TNF-α)	81	9%	0%	0%	23%

a For each probeset the total number of samples was either 60 or 81. The U133A does not contain all of the probesets from the U133Plus2.0. U133A data was used for 15 "Normal" and 6 "Transplant" samples.

**Table 2:** Cytokine genes examined by microarray and rt-PCR in Mayo Clinic kidney transplant specimens.

				**Microarray detection**			**rt-PCR detection**		
	Gene Name	Probe Set ID	BLAST of Consensus Probe Sequence OK?	%P of Mayo samples (n = 30)	Min Detection p-value	Max Detection p-value	%Detected in Mayo samples (n = 29)	Min Ct	Max Ct
1	TNF-α	207113_s_at	Yes	0.0%	0.0806	0.3989	100%	30.03	36.48
2	IFNγ	210354_at	Yes	0.0%	0.0562	0.5676	100%	32.97	41.78
3	IL-6	205207_at	Yes	70.0%	0.0007	0.1116	100%	30.83	39.89
4	IL-10	207433_at	Yes	0.0%	0.1116	0.6963	100%	32.82	37.16
5	TGF-β 1								
		203085_s_at	Yes	0.0%	0.1497	0.9324	100%	26.55	32.54
		203084_at	Yes	0.0%	0.1497	0.6338	100%	26.55	32.54
6	FoxP3								
		224211_at	Yes	0.0%	0.2742	0.8286	100%	30.87	37.22
		221333_at	**NO**^a^	0.0%	0.6655	0.9539	-	-	-
		221334_s_at	**NO**^a^	0.0%	0.0806	0.6655	-	-	-
7	IFNγ-R1								
		202727_s_at	Yes	100.0%	0.0002	0.0020	100%	28.53	33.95
		242903_at	**NO**^b^	100.0%	0.0002	0.0376	-	-	-
		211676_s_at	Yes	96.7%	0.0007	0.0676	100%	28.53	33.95

a Contains probesets for the FoxP3 gene which overlap with the JM2 gene.

b Contains probesets for the IFNγ-Receptor 1 gene that specifically bind the DNA sequence of the gene.

**Table 3:** Comparisons of changes in gene expression determined by microarray and rt-PCR in Mayo Clinic kidney transplant specimens.

		**RT-PCR**		**Microarray**			
**Transcript**	**Analysis**	**Fold change**	**p-value**^a^	**Fold change**	**p-value**	**%P**	**Acceptable correlation**^b^
**A. Undetectable by microarray**							
TNFα							
	TxNormal (0–12)	1.62	0.288	-1.05	0.051	***0%***	***No***
	TxFibrosis (0–12)	2.05	0.061	-1.01	0.728	***0%***	***No***
IFNγ							
	TxNormal (0–12)	2.72	0.172	-1.04	0.152	***0%***	***No***
	TxFibrosis (0–12)	3.11	0.028	1.02	0.537	***0%***	***No***
IL-10							
	TxNormal (0–12)	1.54	0.271	-1.05	0.058	***0%***	***No***
	TxFibrosis (0–12)	3.02	0.000	-1.05	0.134	***0%***	***No***
TGFβ_1_							
	TxNormal (0–12)	2.53	0.003	-1.11	0.012	***0%***	***No***
	TxFibrosis (0–12)	2.93	0.000	-1.09	0.002	***0%***	***No***
Fox-P3							
	TxNormal (0–12)	2.56	0.030	-1.07	0.029	***0%***	***No***
	TxFibrosis (0–12)	2.33	0.000	-1.13	0.001	***0%***	***No***
**B. Detectable by microarray**							
GRZMA							
	TxNormal (0–12)	7.25	0.021	1.32	0.072	42%	***No***
	TxFibrosis (0–12)	9.21	0.000	2.06	0.000	50%	Yes
IL-6							
	TxNormal (0–12)	-12.50	0.029	-1.26	0.008	79%	Yes
	TxFibrosis (0–12)	-7.69	0.005	-1.23	0.000	63%	Yes
FN1							
	TxNormal (0–12)	2.84	0.001	2.78	0.020	93%	Yes
	TxFibrosis (0–12)	2.50	0.002	2.01	0.001	94%	Yes
IFNγ-R1							
	TxNormal (0–12)	1.45	0.072	1.35	0.061	100%	Yes
	TxFibrosis (0–12)	1.63	0.001	1.37	0.008	100%	Yes
IER3							
	TxNormal (0–12)	-2.80	0.016	-2.54	0.000	100%	Yes
	TxFibrosis (0–12)	-2.24	0.005	-1.70	0.000	100%	Yes
STAT2							
	TxNormal (0–12)	1.72	0.015	1.40	0.005	100%	Yes
	TxFibrosis (0–12)	1.88	0.016	1.43	0.001	100%	Yes
COL1A1							
	TxNormal (0–12)	8.89	0.037	5.82	0.005	100%	Yes
	TxFibrosis (0–12)	8.76	0.001	5.34	0.000	100%	Yes
STAT1							
	TxNormal (0–12)	2.06	0.001	1.89	0.000	100%	Yes
	TxFibrosis (0–12)	2.80	0.002	2.19	0.000	100%	Yes
VCAM1							
	TxNormal (0–12)	1.42	0.168	1.34	0.254	100%	Yes
	TxFibrosis (0–12)	2.43	0.000	2.00	0.000	100%	Yes
PSMB8							
	TxNormal (0–12)	1.78	0.019	1.40	0.041	100%	Yes
	TxFibrosis (0–12)	2.82	0.000	1.83	0.000	100%	Yes

a Calculated by TTEST (equal variance, 2-tailed distribution).

b To be considered "acceptable" both the direction of change (up/down- regulation) and significance (p < 0.05 or p > 0.05) must be the same.
